# The preparedness level of final year medical students for an adequate medical approach to emergency cases: computer-based medical education in emergency medicine

**DOI:** 10.1186/1865-1380-7-3

**Published:** 2014-01-03

**Authors:** Akan Karakus, Nurettin Şenyer

**Affiliations:** 1 Department of Medical Education, Medical Faculty, Ondokuz Mayıs University, 55139 Kurupelit/Samsun, Turkey; 2 Department of Computer Engineering, Ondokuz Mayıs University School of Engineering, 55139 Kurupelit/Samsun, Turkey

**Keywords:** Computer-based simulation, Emergency medicine, Forensic medicine, Medical education

## Abstract

**Background:**

We aimed to observe the preparedness level of final year medical students in approaching emergencies by computer-based simulation training and evaluate the efficacy of the program.

**Methods:**

A computer-based prototype simulation program (Lsim), designed by researchers from the medical education and computer science departments, was used to present virtual cases for medical learning. Fifty-four final year medical students from Ondokuz Mayis University School of Medicine attended an education program on June 20, 2012 and were trained with Lsim. Volunteer attendants completed a pre-test and post-test exam at the beginning and end of the course, respectively, on the same day.

**Results:**

Twenty-nine of the 54 students who attended the course accepted to take the pre-test and post-test exams; 58.6% (n = 17) were female. In 10 emergency medical cases, an average of 3.9 correct medical approaches were performed in the pre-test and an average of 9.6 correct medical approaches were performed in the post-test (t = 17.18, *P* = 0.006).

**Conclusions:**

This study’s results showed that the readiness level of students for an adequate medical approach to emergency cases was very low. Computer-based training could help in the adequate approach of students to various emergency cases.

## Background

There is growing scientific concern about web-based medical simulation systems and many studies on this issue are published each year worldwide. In spite of improvements in medical education, the use of such systems in developing countries is relatively low. However, the rise in public attention to malpractice cases among hospitalized patients has compelled medical educators to review the education system as a whole. Further, the development of new web-based simulation training programs for clinical medicine is challenging the gap between the classic education system and clinical practice in different branches of medical science [1-11]. Results of a growing number of studies conclude that the experiences of medical students improved by using computer-based simulation systems [12-16]. Because of reductions in education costs, the ease of repetition of training courses, and the achievement of adequate treatment response to different medical situations, computer-based medical simulation systems are standard in some developed countries [14-21].

This paper aims to observe the preparedness level of final year medical students in approaching real life emergencies and to evaluate the efficacy of computer-based simulation training in medical education.

## Methods

### Study design

We evaluated the success of the computer-based program Lightening simulation (Lsim) in developing the skills and abilities required of a health professional. Fifty-four final year medical students attended an education course and were trained with the Lsim program in Ondokuz Mayis University School of Medicine on June 20, 2012. Only 29 of the students accepted to take pre-test and post-test exams using the same cases in order to evaluate the preparedness level of students to approach real life emergency cases and to assess the efficiency of the course.

### Specifications of the Lightening simulation (Lsim) program

Lsim is a computer-based prototype simulation program designed to teach medical students and health professionals advanced skills in various medical fields. Learning from mistakes is a common and useful practice in education. Therefore, forensic medicine experiences were taken into account when designing scenarios for the virtual cases. The forensic cases that were not adequately treated according to standard medical protocols became the focus in the scenarios. The scenario development process depended on both standard emergency medicine algorithms and evaluation of malpractice cases.

There are various kinds of computer-based simulation programs designed for medical education worldwide. The most widely known web-based medical simulation training systems based on case scenarios are VpSim [17], openLabyrinth [18], CASUS [19], CAMPUS [20], and Web-SP [21]. Programs like VpSim and openLabyrinth allow branching to provide the student with an opportunity to choose among the different paths of the case, making these programs more realistic. The CASUS, CAMPUS, and Web-SP programs flow independently from the student’s choices. OpenLabyrinth is the only open source program, although it requires licensed products.

In designing our web-based simulation program, we used PHP v5.3.3 & MySQL v5.1.49 software (GPL licensed), a Debian Squeeze/Sid platform, and Fat-free framework v2.0.8. The data of the cases was recorded in a central server.

**Figure 1:**
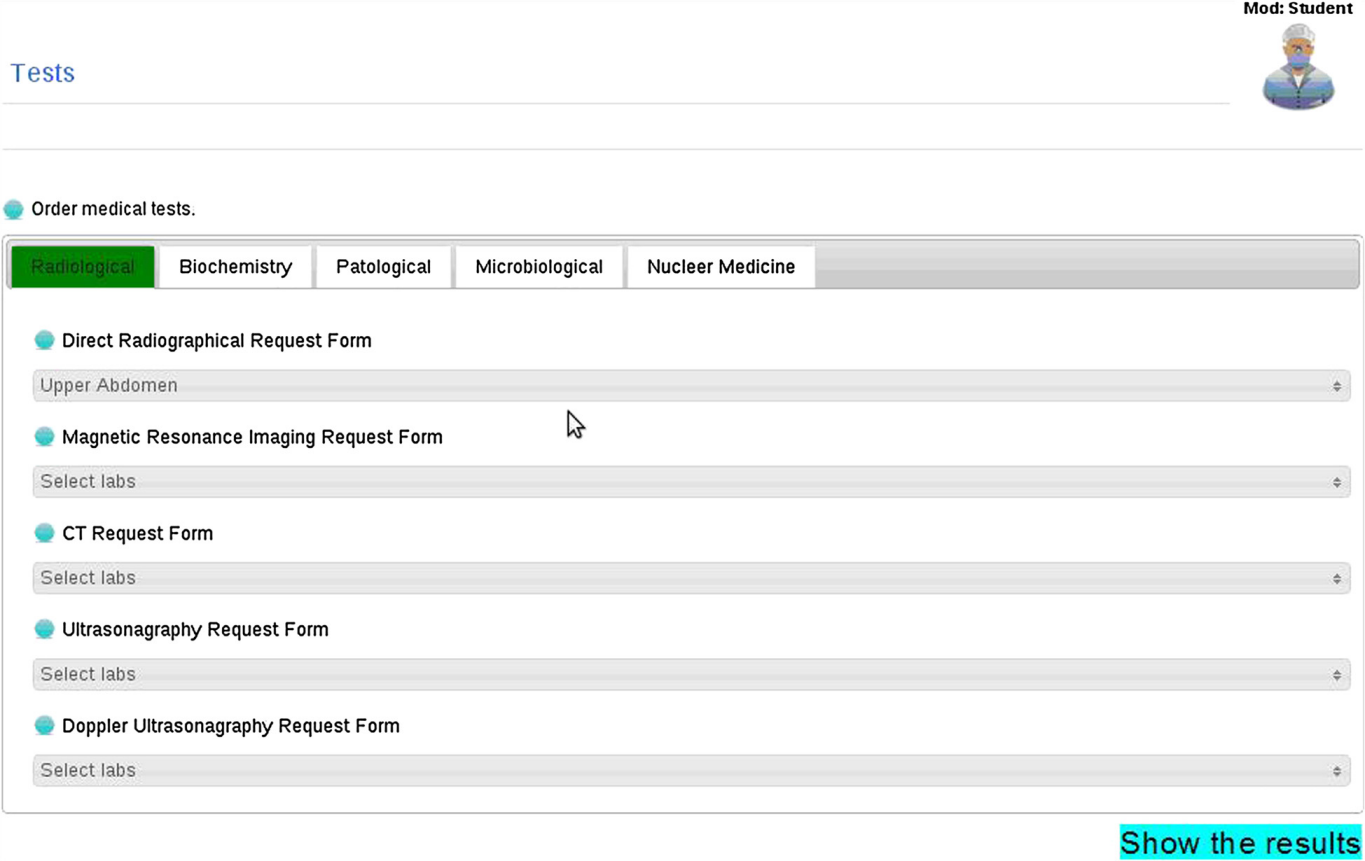
Laboratory and radiologic tests that can be requested and then assessed by the student.

The virtual medical cases are made up of interconnected nodes each representing a part of a medical case such as in the VpSim program [17]. Lsim includes medical approach steps to a patient in different phases. The user must perform an adequate algorithmic medical approach to the virtual patient in order to be successful. The program allows multidimensional medical approaches to a virtual patient (history, physical examination, requesting laboratory and radiologic tests, and drug administration and prescription). The user can request necessary laboratory and diagnostic techniques from a list, which includes tests still in use in a university hospital (Figure 1).

**Figure 2:**
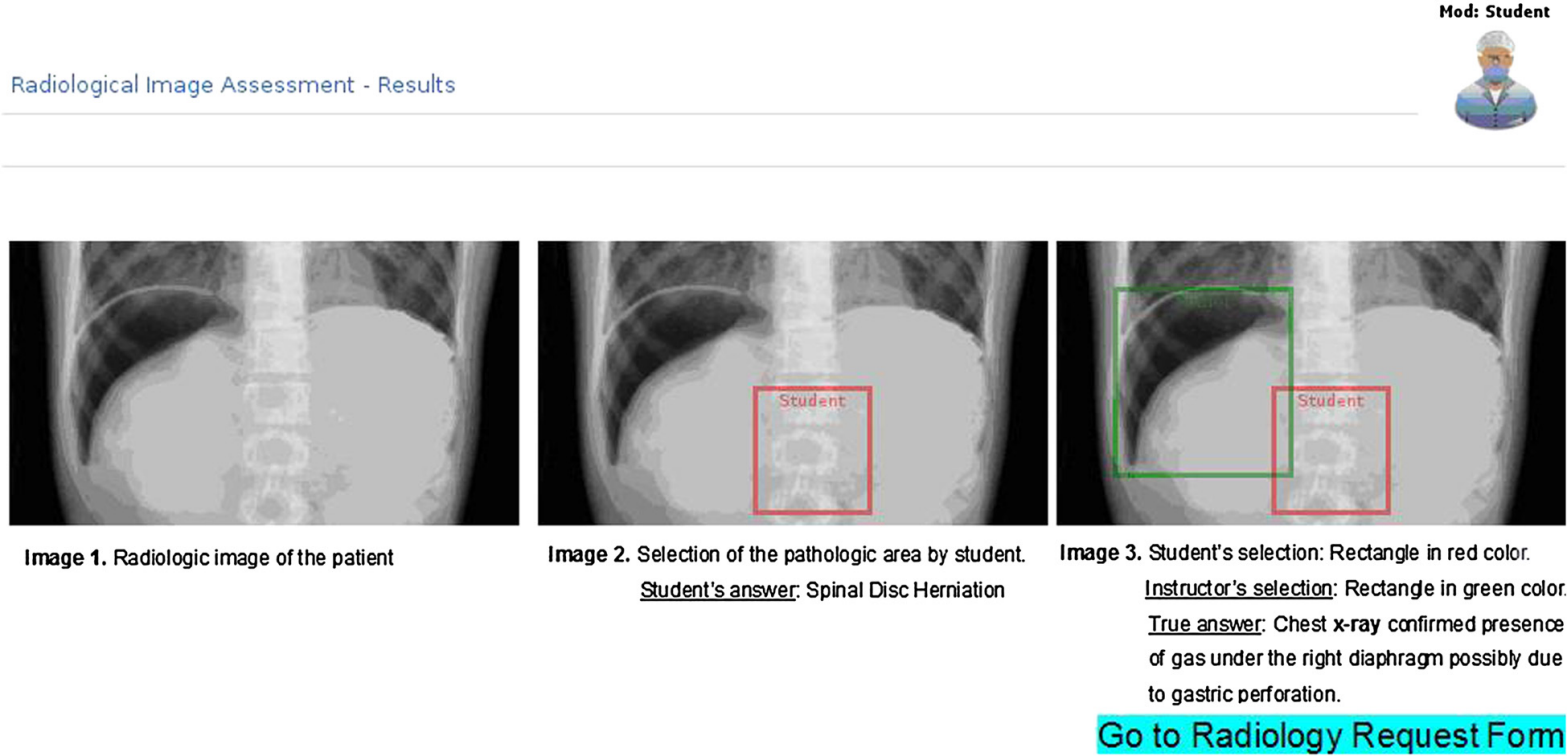
**Assessment of the requested diagnostic image by the student.** The X-ray display was used in case 1.

After the diagnostic images are assessed, the pathology seen on the radiologic film must be shown by the user (Figure 2). The “laboratory and diagnostic tests” ordering screen makes this program more realistic than other simulation programs [17-21].

When diagnosis of the patient is complete, the treatment step begins. In the treatment step, medical advice is provided and, if necessary, the adequate drugs are prescribed. The success of the user in each medical approach step is shown at the end of the simulation.

### Case scenarios

In case 1 (gastric perforation), we scored the case as successful if the student answered the first and second questions correctly. In the first question, the student had to think about the diagnosis of gastric perforation in a female geriatric patient who had presented at the emergency clinic with tenderness and pain in her abdomen and had a medical history of non-steroid anti-inflammatory drug administration for 4 years. In the second question, following evaluation of the patient’s chest radiograph, the student had to show the presence of gas under the right diaphragm due to gastric perforation.

Case 2 (pesticide poisoning) was scored as successful if the student performed all medical approach steps required to treat the poisoning case, which occurred in a green house, such as the removal of clothing, skin decontamination, respiratory support, and antidote administration.

In the emphysema case (case 3), the student had to correctly evaluate the medical history and find a pleural effusion and other signs in the patient’s chest radiograph, as occurs with emphysema.

Life-threatening blood loss may be caused by a stab injury affecting any region of the great vessels. Case 4 presented a stab wound injury of the leg; the case was scored as successful if the student correctly evaluated the medical history, examined the pulses of femoral, popliteal, tibialis posterior, and dorsalis pedis region, and chose the adequate radiologic test (Doppler ultrasonography or computed tomography (CT) of the lower extremity) to assess great vessel injury.

In case 5 (deep vein thrombosis and pulmonary embolism), we scored the case as successful if the students adequately evaluated the medical history and clinical findings, decided on the diagnosis by choosing Doppler ultrasonography of the deep venous system, and if they chose CT of the chest with intravenous contrast, which is accepted as the principal imaging test for the diagnosis of pulmonary embolism.

In case 6 (spontaneous dissection of the aorta) and case 7 (traumatic dissection of the aorta), we scored the cases as successful if the student adequately evaluated the medical history (factors related to degenerative aortic aneurysms such as aging, cigarette smoking, obesity), clinical and radiological findings (such as widening of the mediastinal shadow and displacement of the trachea), and decided on the diagnosis by choosing transesophageal echocardiography, contrast-enhanced CT or magnetic resonance imaging (MRI). Students also needed to initiate medical therapy to control hypertension as soon as the diagnosis was considered.

In post-accident rib fractures and subsequent hemothorax (case 8), we scored the case as successful if the student adequately evaluated the medical history, clinical and radiological X-ray findings, and decided on the diagnosis by choosing CT or MRI. In addition, tube thoracostomy treatment should have been implied by the student, which allows continuous quantification of bleeding.

In retroperitoneal hematoma (case 9), we scored the case as successful if the student adequately evaluated the trauma history, laboratory findings (such as decreasing of blood hemoglobin level in time), and decided on the diagnosis by choosing CT or MRI.

In meningococcemia (case 10), we scored the case as successful if the student adequately evaluated the medical history, clinical findings (such as high body temperature and the prominence of erythematous papular hemorrhagic skin lesions seen on the arm of the patient on screen) and laboratory findings, decided on the diagnosis of meningococcemia, and preferred a third-generation cephalosporin for initial therapy.

### Measures and data analysis

Volunteer attendants completed a pre-test before initiation of the course and they completed a post-test exam with the same questions at the end of the course on the same day. Pre- and post-test results were interpreted based on current emergency medicine guidelines. A student was successful if he or she diagnosed the disease and suggested the correct treatment approach. The independent sampling test and Pearson’s χ^2^ test were used to analyze the data. Fisher’s exact test was carried out when necessary. Statistical significance was accepted at *P* <0.05.

## Results

The total number of students in the study was 29; 58.6% (n = 17) were females. The mean age (±SD) of all attendants was 23.96 ±0.70. The mean ages (±SD) of the female and male students were 23.7 ±0.7 and 24.2 ±0.6, respectively.

We found that in the 10 medical cases, an average of 3.9 correct medical approaches were carried out in the pre-test and an average of 9.6 correct medical approaches were performed in the post-test (t = 17.18, *P* = 0.006) examinations.

In all emergency cases, the computer-based simulation training method was found to be effective in teaching adequate medical approaches to emergency cases (Table 1). The simulated cases included gastric perforation (case 1), pesticide poisoning (case 2), emphysema (case 3), stab wound injury to femoral vein (case 4), deep vein thrombosis and pulmonary thrombus (case 5), spontaneous dissection of aorta (case 6), traumatic dissection of aorta (case 7), post-traumatic rib fractures and subsequent hemothorax (case 8), retroperitoneal hematoma (case 9), and meningococcemia (case 10). The computer-based medical training was particularly successful in the infectious disease cases (cases 3 and 10) (Table 1).

**Table 1:** Comparison of rates of accurate medical approaches done before and after computer-based simulation training (post-test and pre-test results are shown)

**Cases**	**Post-test n (%) success**	**Pre-test n (%) success**	**OR (odds ratio)**	**CI (95%)**	***P***
Case 1	28 (96.6)	13 (44.8)	18.5	7.3–46.5	<0.0001
Case 2	28 (96.6)	12 (41.4)	39.7	20.3–77.7	<0.0001
Case 3	28 (96.6)	17 (58.6)	19.8	2.4–165.8	<0.0001
Case 4	26 (89.7)	8 (27.6)	22.8	5.4–96.6	<0.0001
Case 5	26 (89.7)	7 (29.2)	24.8	5.7–108.0	<0.0001
Case 6	28 (96.6)	10 (34.5)	53.2	5.4–96.6	<0.0001
Case 7	28 (96.6)	2 (6.9)	378.0	32.4–4415.7	<0.0001
Case 8	27 (93.1)	13 (44.8)	16.6	3.3–83.2	<0.0001
Case 9	29 (100)	22 (75.9)	18.5	7.3–46.5	<0.0001
Case 10	29 (98.3)	20 (69.0)	26.1	10.4–65.4	<0.0001

## Discussion

In medical education, one of the most important aspects is the preparation of medical students for real life scenarios. The present study aimed to observe the preparedness level of final year medical students in their medical approach to emergency cases and evaluate the effectiveness of computer-based simulation training. The overall adequate medical approach rate in emergency cases was very low (3.9 of 10 cases) in pre-test evaluation. After simulation training, the success rate increased to 9.6 in 10 cases.

In medicine, appropriate diagnosis is of paramount importance for adequate treatment. In light of our experience, it is imperative that medical practitioners learn new guidelines issued for diagnosis and treatment algorithms of various diseases before and after graduation from medical school [22]. It has been stated that hemoglobin drops within minutes of a traumatic injury and therefore predicts the need for an intervention to stop a hemorrhage [23]. As in our virtual cases of stab wound injury to the femoral region (case 4), post-traumatic rib fractures and subsequent hemothorax (case 8), retroperitoneal hematoma (case 9), monitoring of patients’ hemoglobin levels is important to assess a lethal hemorrhage. After computer-based training, the preparedness of final year medical students for an adequate approach to these cases was increased.

In our opinion, in order to lower the frequency of inadequate treatment, it is important to increase practitioners’ abilities and skills. This would not only protect patients’ health but would also decrease health care costs. It is emphasized that forensic autopsies might be a supportive practice in anatomy education [24]. Nevertheless, the scientific evidence arising from forensic autopsies could also be used in clinical medical education.

It has been discussed that teaching and learning through medical errors induces an emotional impact on the student; the use of simulation may help by learning from near misses and others’ errors [25]. Evaluating malpractice cases and learning how to avert them, is a feasible approach for improving the standards in health care. Thus, common medical errors could be eliminated. The Lsim program has the capability to instantly evaluate the students’ medical approaches by performing online examinations. Through this program, students can realistically order all laboratory and radiological tests required, evaluate the test results and radiologic images, plan the medical approach, and provide adequate treatment to the patient such as in a real life scenario.

## Conclusions

It is judicious that an increase in medical professionals’ abilities and skills would decrease the frequency of malpractice cases due to the inadequate treatment of patients. Computer-based medical simulation is efficient in testing the knowledge level of students and in increasing the success rate in performing the adequate medical approach to emergency cases. In order to save patient lives and to decrease the number of medical errors, next generation simulation-training systems should be the standard in medical education.

## Competing interests

The authors declare that they have no competing interests.

## Authors’ contributions

In the present study, two-center collaboration on developing a web-based medical simulation program for preventing medical errors was designed. Medical educational design of the work was principally carried out by MD Dr AK. The computer programming phase of the work was principally carried out by PhD Dr NŞ. The Lsim program is a registered trademark in Turkey. The paper was prepared collaboratively by the authors. No funding was received for the present study. The two authors were principal investigators of this study. Both authors read and approved the final manuscript.

## Authors’ information

Two-center collaboration on developing and testing a computer-based medical education simulation program for medical education.
